# Interface-Enhanced Mg/PLA Composite with Superior Mechanical, Biodegradable and Biocompatible Properties for Orthopedic Implants

**DOI:** 10.3390/jfb17050210

**Published:** 2026-05-01

**Authors:** Wenchen Teng, Zhuoyi Wang, Ziyue Xu, Jie Xin, Chao Sun, Yi Shao, Cheng Wang, Chenglin Chu, Feng Xue, Jing Bai

**Affiliations:** 1 Jiangsu Key Laboratory of Advanced Metallic Materials, School of Materials Science and Engineering, Southeast University, Nanjing 211189, China; tengwch@163.com (W.T.); trailsoflion818@163.com (Z.W.); 230249093@seu.edu.cn (Z.X.); 220242778@seu.edu.cn (J.X.); sunchao_239@163.com (C.S.); sy@i-bmd.org (Y.S.); cheng.wang@seu.edu.cn (C.W.); clchu@seu.edu.cn (C.C.); 2 Institute of Biomedical Devices (Suzhou), Southeast University, Suzhou 215163, China

**Keywords:** composite, mechanical properties, surface modification, mechanical interlocking

## Abstract

Magnesium (Mg) reinforced polylactic acid (PLA) composites have attracted increasing interest for orthopedic implants to solve the insufficient strength of PLA and to utilize the bioactive advantages of Mg ions in promoting bone formation. However, the weak interfacial adhesion between the Mg and PLA limits the applications of the composite. In this study, a dual interfacial enhancement approach was designed to combine surface fluorination with perforation. During hot pressing, molten PLA infiltrates the pores to form a ‘rivet-like’ mechanical interlocking. This structure significantly alters the load transfer and degradation behaviors of the composite. Compared to pure PLA, the dual treatment significantly elevated the bending strength by 49%, alongside an increase in the bending strain from 15% to 25%. Moreover, in vitro degradation tests revealed that this strategy suppresses H_2_-induced delamination, and stabilizes both pH and Mg^2+^ release. Consequently, the bending strength remained at 86% after six weeks of in vitro degradation. In addition, the composite exhibits excellent biocompatibility, with MC3T3-E1 cell viability exceeding 90% in 100% extract. These results demonstrate that the reinforced Mg/PLA composite exhibits excellent mechanical properties, degradation stability, and biocompatibility, showing high potential for load-bearing orthopedic fixation applications.

## 1. Introduction

Biodegradable polymer implants have attracted growing interest for the advantages of gradual degradation and absorbability by the human body, thereby sparing patients from secondary surgery [1,2,3]. Representative polymers, including polycaprolactone (PCL), poly (lactic-co-glycolic acid) (PLGA), and polylactic acid (PLA), demonstrate favorable safety profiles, low toxicity, and adaptability for application requiring significant material flexibility [4,5,6]. Among these materials, PLA is the most widely used biodegradable polymer due to its excellent biocompatibility and processability. Moreover, PLA has been recognized as safe and has been approved by the U.S. Food and Drug Administration (FDA) [7]. However, PLA exhibits certain limitations, including inadequate mechanical properties, and the production of acidic degradation products generated by PLA can cause local inflammatory reactions. Furthermore, the degradation rate of PLA lags behind the pace of osteogenesis, which severely limits its application in load-bearing orthopedic implants [8,9].

To overcome the shortcomings of PLA, researchers have explored various approaches, such as blending it with other polymers [10] or developing composite materials [11]. Synergizing bioactive metals or ceramics within polymer matrices has emerged as a pivotal strategy to bypass the limitations of single-component materials. Magnesium (Mg) and its alloys are considered to be especially promising reinforcements for PLA-based systems [12,13]. Mg stands out as an ideal choice for orthopedic applications, owing to its bone-mimetic modulus, promoting bone healing, and innate biodegradability [14]. Extensive studies have shown that incorporating Mg particles, fibers, or sheets into PLA markedly boosts both the mechanical properties and the bone-forming capabilities of the resulting material [15]. For instance, Lee et al. [16] reported that adding Mg particles and TCP microspheres to PLA substantially enhanced the mechanical properties and bioactivity of the composite. The rapidly degrading Mg particles promote pore formation, and the released Mg^2+^ ions contribute to bone regeneration. The increasing local pH enhanced the antibacterial effects. Zhang et al. [17] demonstrated that creating a layered configuration by integrating dual PLA films with an AZ91 surface treated with fluoride significantly improves both the load-bearing capacity and rigidity. This innovative design proves particularly effective in preserving the mechanical stability of dental implants during initial recovery phases.

Nevertheless, establishing the stable interface bonding between Mg and PLA remains a major challenge. During degradation of the Mg/PLA composites, Mg generates H_2_ that gets trapped at the interface and weakens the bond, ultimately causing delamination and early stage failure of the composite and hindering clinical translation [18]. To overcome the interfacial weaknesses, Cai et al. [19] improved the interface bonding between Mg fibers and PLA matrix by hot extrusion and cold drawing processes. This treatment slowed down the rapid degradation of internal Mg fibers and achieved controlled release of Mg^2+^. Additionally, Abdeljawad et al. [20] enhanced the compatibility and cell adhesion between Mg and PLA through a two-polymer block copolymer as an interfacial compatibilizer.

In this study, we employ a dual enhancement strategy by combining fluorination and perforation. We constructed a robust ‘rivet-like’ mechanical interlocking at the boundary of the Mg sheet and the PLA matrix. Finite element modeling guided the spatial design of these interlocking sites to ensure the maximum load transfer. By comprehensive mechanical properties experiments and in vitro degradation testing, this approach was systematically verified to improve mechanical retention and biocompatibility.

## 2. Materials and Methods

### 2.1. Materials Preparation

#### 2.1.1. PLA Preparation

PLA (3051D, Nature Works, Minnetonka, MN, USA), with an average molecular weight (Mn) of approximately 150,000 and a melting point of about 179 °C, was used. PLA films were prepared via solvent evaporation. The PLA/dichloromethane solution (0.125 g/mL) was cast into a tray and evaporated in a fume hood for 24 h, yielding 0.5 mm thick films. These were cut into 60 mm × 12 mm specimens and dried completely at 50 °C for 6 h.

#### 2.1.2. Mg Sheet Preparation and Surface Treatment

AZ31 Mg alloy sheets (60 mm × 10 mm × 1 mm), prepared by rolling and wire cutting, were used to prepare the Mg/PLA composites. The AZ31 sheets were fluorinated by immersion in a 40% hydrofluoric acid (HF) solution at room temperature for 24 h, rinsed with anhydrous ethanol, and air dried. Laser perforation was carried out using a laser marking machine (Shanghai Shenwei Electronic Technology Co., Ltd., Shanghai, China) at 100% power and at a speed of 200 mm/s. Circular holes with a diameter of 1.5 mm were carved to serve as mechanical interlocking sites. The carved sheets were subsequently polished using progressively finer sandpapers, ultrasonically cleaned in ethanol, and then dried.

#### 2.1.3. Mg/PLA Composite Fabrication

**Figure 1:**
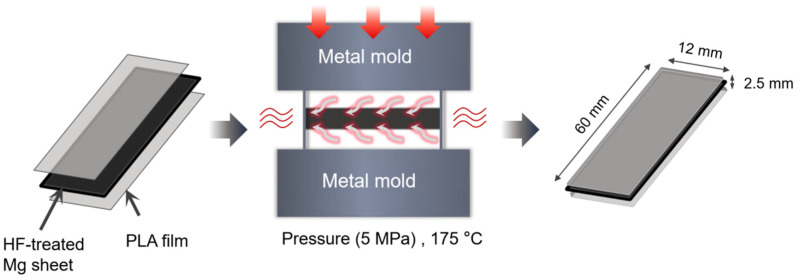
Schematic diagram of the hot pressing fabrication process for the Mg/PLA composite.

The fabrication process of the Mg/PLA composite via hot pressing is schematically illustrated in Figure 1. First, PLA films were placed on both sides of the HF-treated Mg sheet to form a sandwich structure. This assembly was then wrapped in aluminum foil coated with a dry release agent and placed into a metal mold. The hot pressing was conducted at 175 °C under a pressure of 5 MPa for 15 min. This process yielded a composite sheet with dimensions of 60 mm × 12 mm × 2.5 mm. The composites were classified into three groups according to the surface treatment applied to the Mg sheet: untreated (Mg/PLA), fluorinated-only (Mg-F/PLA), and fluorinated with laser perforation (Mg-F-C/PLA).

### 2.2. Finite Element Modeling of Bending Behavior

Under unidirectional static loading conditions, failure in the Mg/PLA composite sheet generally commences through matrix cracking and interfacial delamination, resulting in a progressive decline of the mechanical properties. The ‘Hashin criterion’ [21] was selected as the damage criterion. ABAQUS was used to simulate the bending response, with the Mg alloy modeled as an isotropic hardening material following the von Mises plasticity model [22]. The detailed material parameters for PLA and AZ31 used in the simulation are summarized in Table 1.

**Table 1:** Material Parameters of PLA and AZ31.

PLA		AZ31	
Parameter	Value	Constitutive parameter	
Elastic modulus/GPa	3	Yield stress/MPa	Plastic strain
Poisson’s ratio	0.37	175.13	0
Density/(g/cm^3^)	1.25	198.51	0.0082
Tensile strength (MPa)	60	213.31	0.0142
Compression strength (MPa)	80	225.01	0.0201
Fracture toughness/(mJ·mm^−2^)	1.5	251.88	0.0381
		264.71	0.0505
AZ31		270.16	0.0569
Parameter	Value	279.54	0.0703
Elastic modulus/GPa	44	283.76	0.0772
Poisson’s ratio	0.35	291.08	0.0918
Density/(g/cm^3^)	1.83	297.43	0.1074
Elongation/%	6	302.96	0.1234
Yield strength/MPa	175	308.78	0.1474

The composite laminate was discretized by C3D8 solid elements. A global element size of 0.4 mm was applied, with local refinement to 0.2 mm within the carved regions. The PLA layer was treated as isotropic, and the Mg/PLA interface was tied with a Tie constraint. The punch and two supports were modeled as discrete rigid bodies in hard contact with the laminate, incorporating a tangential friction coefficient of 0.3. The simulation replicated the three-point bending setup with a loading speed of 2 mm/min over 12 min, comprising 1000 increments.

To evaluate the contribution of the mechanical interlock, simulations were conducted with laser-perforated area fractions ranging from 0% to 20%. The number of carved holes was fixed at five, spaced 10 mm apart, and the area fraction was adjusted by modifying the radius of the holes (0–2.76 mm).

### 2.3. Mechanical Property Test

The bending strength of the Mg/PLA composite with different surface treatments was evaluated by a universal testing machine (SANS Testing Equipment Co., Ltd., Shenzhen, China). The testing procedure was performed in accordance with the ASTM D7264 standard [23], with specimen dimensions of 60 mm × 12 mm × 2.5 mm, a span length of 40 mm, and a loading rate of 2 mm/min. Fracture was defined as the point at which the load dropped to 50% of its maximum value. The bending strength (σ_f_) was calculated using the following equation:

$$\sigma_{f}=\frac{3PL}{2bh^{2}}$$

where σ_f_ is the flexural strength (MPa), P is the maximum load at failure (N), L is the span length during three-point bending (mm), b is the specimen width (mm), and h is the specimen thickness (mm).

### 2.4. In Vitro Degradation Test

To simulate the physiological conditions, in vitro degradation trials were conducted by Hank’s balanced salt solution. Composite specimens with different surface treatments were selected, and each sample was immersed in Hank’s solution at a mass-to-liquid ratio of no less than 1 g per 30 mL. The degradation periods were set at 1, 2, 4, and 6 weeks, with the degradation temperature maintained at 37 °C. Prior to the immersion, the medium’s pH was calibrated to 7.4. During the degradation process, the pH of the solution was measured weekly, maintaining a distance of approximately 1 mm between the pH probe and the sample surface. The Hank’s solution was refreshed once per week. At each predetermined degradation time point, samples were retrieved, rinsed thoroughly, and dried prior to flexural strength testing. Additionally, the Mg ions released in the extract were determined by inductively coupled plasma optical emission spectrometry (ICP-OES, SPECTROBLUE, SPECTRO Analytical Instruments GmbH, Kleve, Germany). Data integrity was maintained by conducting parallel experiments in triplicate and reporting the average findings.

### 2.5. Material Characterizations

Surface morphology was investigated using a Zeiss Crossbeam 350 scanning electron microscope (SEM, Carl Zeiss AG, Oberkochen, Germany). To identify the chemical composition of the Mg alloy and its resultant corrosion layers, an Oxford Instruments Ultim Max 65 energy dispersive spectrometer (EDS, Oxford Instruments, Abingdon, UK) was employed. Following the initial observation, the corrosion products were stripped using a standard solution of CrO_3_ for 5 min at room temperature, allowing for the subsequent SEM inspection of the cleaned substrate.

Phase analysis was performed by X-ray diffraction (XRD, Bruker D8-Discover, Billerica, MA, USA). Diffraction patterns were recorded over a 2θ range of 10–90°, with a scan rate of 5°/min. The XRD patterns were analyzed by the Jade program.

### 2.6. Cytocompatibility

MC3T3-E1 cells (Cell Bank of the Chinese Academy of Sciences, Shanghai, China) were used to evaluate the cytocompatibility of the samples. The cells were maintained in α-MEM supplemented with 10% FBS and 1% penicillin–streptomycin at 37 ℃ in a humidified atmosphere of 5% CO_2_. To prepare the extracts, UV-sterilized samples were immersed in α-MEM complete medium with a surface area-to-volume ratio of 1.25 cm^2^/mL. After incubation at 37 ℃ for 72 h, the supernatant was collected and stored at 4 ℃ for subsequent experiments. For the cytocompatibility test, MC3T3-E1 cells were initially seeded in 96-well plates and cultured for 24 h to allow attachment. The culture medium was then replaced with the prepared extract solution. After treatment for 3 and 5 days, cell viability and proliferation were evaluated using live/dead staining and MTT assay, respectively. The live/dead staining images were captured by a fluorescence microscope (IX83, Olympus, Tokyo, Japan). The absorbance for MTT was measured at 570 nm with a reference wavelength of 490 nm using a microplate reader (Multiskan GD, Thermo Fisher Scientific, Waltham, MA, USA).

## 3. Results

### 3.1. Bending Performance of the Composite Obtained by Finite Element Modeling

**Figure 2:**
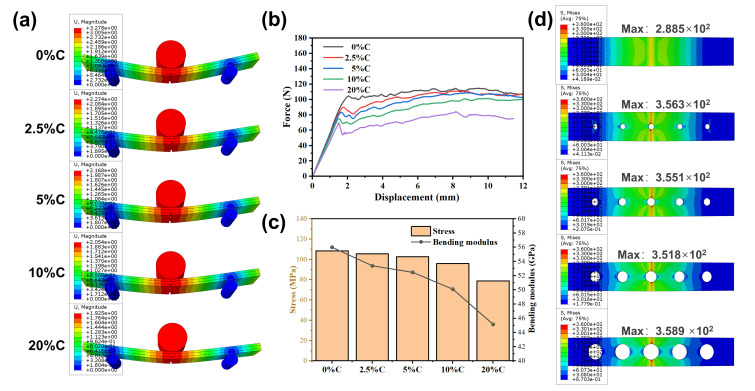
Simulation results of composite sheet: (**a**) displacement cloud diagram at the moment of a complete crack occurrence; (**b**) bending force–displacement curve; (**c**) bending strength and bending modulus; (**d**) stress cloud diagram of Mg sheet at the same displacement (3.27 mm).

The simulation results of the composite sheets with different area fractions are shown in Figure 2. As the hole radius increased, the strain at the crack initiation decreased, indicating earlier crack occurrence (Figure 2a). The load–displacement curves (Figure 2b) revealed that the higher laser-perforated area fractions reduced the bending resistance and strength, with a 20% carved area fraction decreasing the flexural strength to about 70% of that of the uncarved group (Figure 2c). Stress distribution contours at the same displacement (Figure 2d) show that laser perforation induced stress concentration in the Mg alloy, though no significant variation was observed among the different area fractions. Based on these results, a 2.5% area fraction was selected as the optimal design parameter for experimental validation.

### 3.2. Interfacial Microstructure and Mechanical Properties

**Figure 3:**
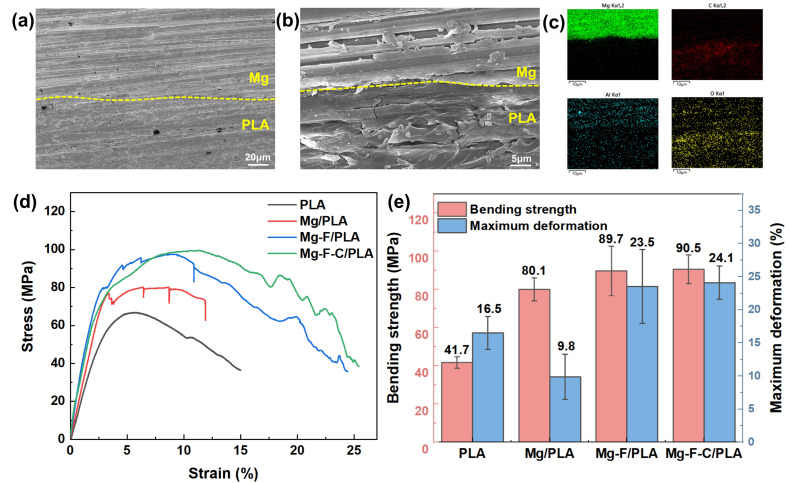
Morphology and mechanical properties of the Mg/PLA composite: (**a**,**b**) SEM images of the Mg/PLA sheet; (**c**) EDS mapping result of (**b**); (**d**) bending stress–strain curves; (**e**) bending strength and maximum deformation of the PLA, Mg/PLA, Mg-F/PLA, and Mg-F-C/PLA sheets.

Figure 3a,b exhibit the cross-sectional morphology of the composite sheets. The interface between the Mg layer and the PLA matrix was well-bonded, manifesting no visible voids or delamination. EDS mapping (Figure 3c) confirms the distinct distribution of Mg within the metallic region and C in the PLA region, providing additional evidence of effective interfacial integration achieved during the hot-pressing process.

The corresponding bending stress–strain curves are shown in Figure 3d. Pure PLA exhibits the relatively low bending strength of 66.8 MPa, with typical elastic, strengthening, and softening stages. The untreated Mg/PLA sheet (30 vol.% Mg) displays an increased bending strength (80.1 MPa), but fails abruptly after the elastic and strengthening stages, resulting in premature interfacial delamination and fracture of the PLA. With the fluorination treatment, the Mg-F/PLA and Mg-F-C/PLA sheets reach higher bending strength of 89.7 MPa and 90.5 MPa, with maximum deformation of 23.5% and 24.1% each. This result reflected the enhanced interfacial bonding and delayed failure.

### 3.3. Cytotoxicity Evaluation of the Composite Sheets

**Figure 4:**
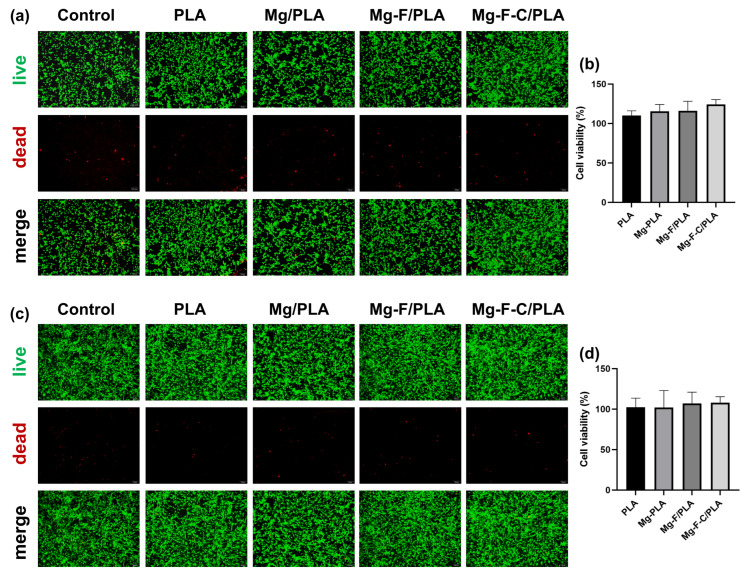
Fluorescence micrographs of MC3T3-E1 osteoblasts cultured in material extracts for (**a**) 3 days and (**c**) 5 days; corresponding cell proliferation rate after (**b**) 3 days and (**d**) 5 days.

Figure 4 presents the live/dead staining and MTT assays of osteoblasts cultured in 100% extracts for 3 and 5 days across five groups: Control, PLA, Mg/PLA, Mg-F/PLA, and Mg-F-C/PLA. At both time points (Figure 4a,b), all groups exhibit predominantly live cells (green) with minimal dead cells (red), indicating high viability and low cytotoxicity. The MTT results (Figure 4b) confirmed cell viability above 80% in all groups with Mg/PLA reaching 108.2% due to Mg^2+^ enhanced proliferation (Figure 4c). After 5 days (Figure 4d), the fluorinated and carved groups (Mg-F/PLA and Mg-F-C/PLA) show viability comparable to the control.

The release of Mg^2+^ during degradation promoted osteoblast growth without inducing cytotoxicity [24,25]. Even the unmodified Mg/PLA maintained a cell viability above 80%, demonstrating good biocompatibility without significant cytotoxicity. Fluorination and patterning further improved corrosion resistance, stabilized pH, and enhanced cytocompatibility, consistent with previous studies [26]. All extracts maintained high cell viability after 5 days confirming the composites’ biocompatibility.

## 4. Discussion

### 4.1. In Vitro Degradation Behavior

Probing the underlying degradation kinetics, we conducted a comparative six-week immersion study to evaluate the microstructure evolution and properties of the Mg-F/PLA and Mg-F-C/PLA composites.

**Figure 5:**
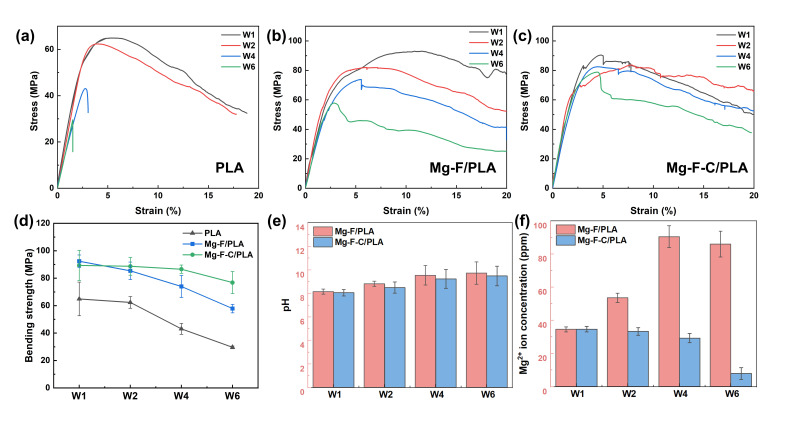
Mechanical property evolution during the degradation: (**a**) PLA sheet, (**b**) Mg-F/PLA sheet, (**c**) Mg-F-C/PLA sheet, and (**d**) evolutions of bending strength. Macroscopic morphology of the Mg-F/PLA and Mg-F-C/PLA sheets: (**e**) pH evolution, (**f**) Mg^2+^ concentration evolution.

Figure 5a exhibits the evolution of bending stress for the PLA, Mg-F/PLA, and Mg-F-C/PLA composite sheets during different degradation periods. Pure PLA remained mechanically stable for the first two weeks, but experienced a sharp decline after four weeks due to ester bond cleavage and reduced polymer molecular weight. After one week of immersion, the Mg-F/PLA sheet exhibited a flexural strength of 92.4 MPa (Figure 5b), slightly higher than that of the Mg-F-C/PLA sheet (89.3 MPa) (Figure 5c). However, after 6 weeks, its strength declined rapidly to 57.9 MPa (only 62.2% of initial), while the Mg-F-C/PLA sheet retained 76.8 MPa (84.9% of initial), indicating superior structural stability. The sustained strength of the composites after 6 weeks is attributed to the Mg sheet’s mechanical support and the self-locking effect with PLA, which delays hydrogen induced gas delamination. For Mg-F/PLA, the curves shifted towards lower strain with increasing degradation, showing rapid post-yield stress drops, that reflect a reduced load-bearing capacity. Conversely, the Mg-F-C/PLA sheet maintained higher yield stress and more stable post-yield behavior, confirming its superior mechanical stability during degradation.

The pH and Mg^2+^ concentration images (Figure 5e,f) show that Mg-F/PLA exhibited a gradual pH rise and Mg ions release. Although the Mg-F/PLA sheet stabilized temporarily between weeks 1 and 2, due to the protective MgF_2_ layer, the concentration of Mg ions increased significantly after 4 weeks. By comparison, the Mg-F-C/PLA composite maintained lower and more stable pH and Mg^2+^ values throughout. The mechanical interlocking structure of Mg-F-C/PLA serves as a physical barrier that slows down the infiltration of corrosive liquids, effectively preventing localized pitting from spreading.

At the early stage of immersion, both composites displayed comparable bending strength, indicating that fluorination alone effectively improved interfacial bonding. However, as degradation progressed, the composites exhibited rapid strength loss accompanied by interfacial cracking, while the Mg-F-C/PLA composite retained about 85% of its initial bending strength after six weeks, showing much better mechanical stability. This contrast indicates that the mechanical interlocking structure effectively constrained interfacial separation and mitigated stress concentration, allowing the composite to better accommodate internal variation during long-term degradation.

**Figure 6:**
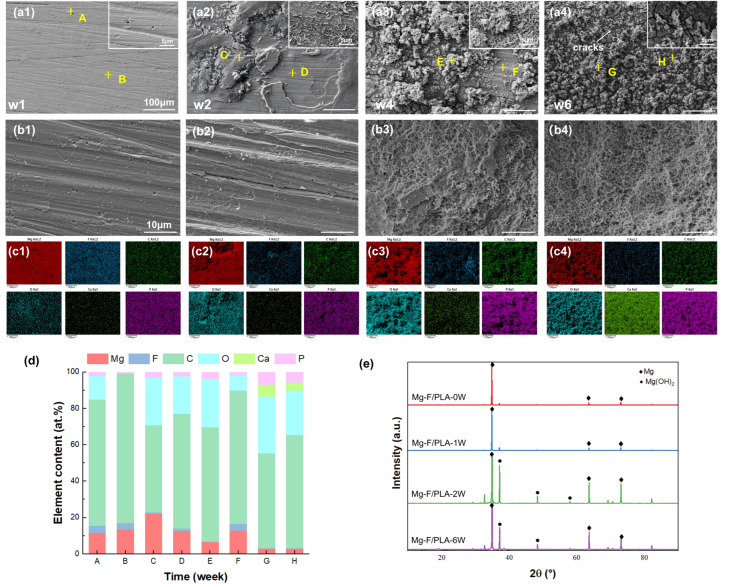
(**a1**–**a4**) Corrosion morphology of Mg-F/PLA sheet; (**b1**–**b4**) morphology of the Mg-F/PLA sheet after removing corrosion products; (**c1**–**c4**) EDS mapping results corresponding to (**a1**–**a4**); (**d**) element analysis of each point in (**a1**–**a4**); (**e**) XRD results of the Mg fluoride sheet.

To further interpret the mechanical properties trends above, surface and corrosion microstructure evolution were examined to correlate the corrosion morphology with the mechanical degradation behavior. Figure 6(a1–a4) present the surface corrosion morphology of the Mg layer in the Mg-F/PLA composite after degradation for 1, 2, 4, and 6 weeks, respectively. Initially, the MgF_2_ layer and PLA effectively blocked Cl^−^ ingress, leaving the surface smooth. Over time, corrosion products accumulated, and by week 6, the Mg surface was fully covered with corrosion products and cracks.

EDS analysis of selected regions (Figure 6(c1–c4),d). A-H indicate dselected EDS analysis points, where A/C/E/G correspond to severely corroded regions and B/D/F/H correspond to mildly corroded regions. The results showed decreasing Mg and F but increasing Ca and P, indicating deposition of Ca–P compounds from Hank’s solution. F was still detectable until week 4 but absent at week 6, suggesting MgF_2_ delamination and loss of protection, accelerating later stage corrosion and supporting its transformation into oxides. A SEM analysis (Figure 6(b1–b4)), after removing corrosion products, revealed a relatively intact Mg layer in the early weeks but increased roughness, porosity, and pitting by weeks 4–6 (Figure 6(b4)). The XRD results (Figure 6e) confirmed the presence of Mg(OH)_2_ with increasing degradation. Minor phases, such as carbonates or phosphates, were not clearly distinguishable, likely due to low content or peak overlap. Future work could employ higher-resolution techniques to identify these phases more precisely.

**Figure 7:**
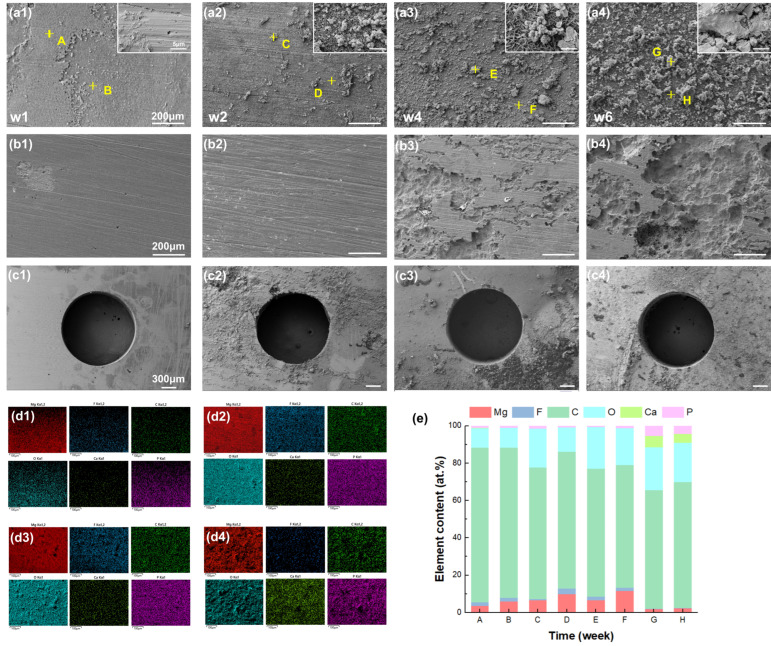
(**a1**–**a4**) Corrosion morphology of Mg-F-C/PLA sheet; (**b1**–**b4**) morphology after removing corrosion products; (**c1**–**c4**) corrosion morphology around the interlocking pores; (**d1**–**d4**) EDS mapping results corresponding to (**a1**–**a4**); (**e**) element analyses of each point in (**a1**–**a4**).

Figure 7(a1–a4) show the surface corrosion morphology of the Mg layer in the Mg-F-C/PLA composite after degradation for 1, 2, 4, and 6 weeks, respectively. Similar to the prior analysis, points from severely (point A/C/E/G) to mildly (point B/D/F/H) corroded areas were examined. After week 1, the surface remained largely smooth; by week 6, significant corrosion product accumulation was observed.

After removal of corrosion products (Figure 7(b1–b4)), the surface remained relatively intact, but showed increasing roughness and porosity from week 4 onward. Figure 7(c1–c4) reveal enhanced localized corrosion around the carved pores, indicating preferential pathways for corrosive media. Nevertheless, the pores remained intact throughout the degradation period, confirming that a good interfacial bonding between Mg and PLA occurred.

The EDS analysis (Figure 7(d1–d4)) exhibited a clear elemental evolution, where the reduction in Mg and F elements and the accumulation of O and C contents are mapped, alongside shifts in the Ca and P distributions over the degradation period. Point scanning (Figure 7e) confirmed that a protective Ca–P layer gradually formed on the surface.

According to the typical Mg alloy corrosion mechanisms [27], the initial MgF_2_ layer inhibits anodic dissolution (Mg → Mg^2+^ + 2e^−^) and hydrogen evolution (2H_2_O + 2e^−^ → H_2_↑ + 2OH^−^). As a result of Cl^−^ ingress and OH^−^ accumulation, the MgF_2_ layer is slowly degraded and delaminated, leading to the observed drop-off in F content. The subsequent exposure of Mg triggers accelerated pitting, uniform dissolution, and H_2_ release, followed by the deposition of Mg(OH)_2_ and Ca–P compounds, finally leading to a total loss of protection [28].

The difference in corrosion morphology further explains the divergence in mechanical properties. The Mg-F/PLA composite primarily suffered from pitting and interfacial delamination, leading to an early loss of integrity [29]. On the other hand, Mg-F-C/PLA exhibited uniform corrosion morphology and remained intact. This transition of local-to-uniform corrosion is attributed to the dual surface modification. The MgF_2_ layer gave an initial chemical barrier, while the PLA infiltrated into the interlocking pores offered a physical barrier to restrict fluid ingress and chloride attack. Together, these effects extended the stability of the protective layer and delayed the severe corrosion process.

The immersion test comparison also showed that the dual-treated composites were superior, as the Mg-F/PLA composite was affected by early pitting and fast degradation, while the Mg-F-C/PLA composite retained higher mechanical stability and more uniform corrosion morphology. It shows that the mechanical interlocking can not only enhance the initial interfacial bonding, but ensure a more reliable long-term performance in physiological conditions.

### 4.2. The Underlying Mechanisms for Interface Enhancement

**Figure 8:**
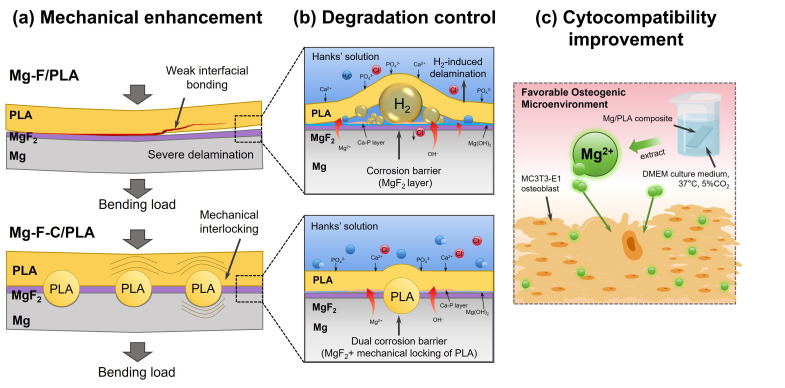
Schematic illustration of the synergistic enhancement mechanisms of the Mg-F-C/PLA composite compared to the Mg-F/PLA composite: (**a**) mechanical enhancement, (**b**) degradation control, (**c**) cytocompatibility improvement.

In this work, we proposed an interfacing double strengthening strategy including fluorination and perforation for improving the interfacial bonding of Mg/PLA composites. The schematic illustration of the synergistic enhancement mechanism of the Mg-F-C/PLA composite relative to that of the Mg-F/PLA composite is summarized in Figure 8. From a mechanical perspective (Figure 8a), the Mg-F/PLA composite exhibits relatively weak interfacial adhesion, which makes the structure prone to interfacial delamination under bending loads. The macroscopic failure of conventional metal–polymer laminates predominantly results from interfacial shear sliding. In the Mg-F/PLA composite, only depending on the chemical affinity of the MgF_2_ layer yields an inherently weak interface. When bending strength surpasses the physical adhesive strength at the interface, abrupt and severe delamination ensues. Conversely, by introducing interlocking carved pores on the Mg surface, the Mg-F-C/PLA composite exhibits a transformed failure mechanism. During hot pressing, molten PLA flows into the pores and solidifies into a robust “rivet-like” mechanical interlocking structure [30]. The introduction of the structure enables the applied load to be evenly distributed, effectively counteracting the shear force and preventing the propagation of cracks that could cause interfacial fracture. [31,32]. The finite element modeling also confirmed that, even though there is a concentration of stresses around the carved areas, the plastic deformation of the PLA and interfacial friction aid in the dissipation of energy, thus, efficiently preventing the initiation and growth of cracks.

Regarding degradation control, the interlocking structure also plays a decisive role in regulating the ion erosion (Figure 8b). The degradation of the Mg alloy will produce an upward H_2_ evolution. For the Mg-F/PLA composite, this local H_2_ escape will forcibly peel the polymer from the Mg alloy, destroying the anti-corrosion protection of the PLA. In addition, the ingress of chloride ions (Cl^−^) typically accelerates substrate dissolution, while calcium (Ca^2+^) and phosphate (PO_4_^3−^) ions precipitate to form the Ca–P layers on the surface of Mg. The combined effect of rapid corrosion product accumulation and H_2_ evolution pressure causes the MgF_2_ layer on the Mg-F/PLA sheet to peel prematurely. By contrast, the Mg-F-C/PLA composite forms a ‘two-layered corrosion barrier’ [33]. PLA filling in the interpenetrated pores act as a physical barrier limiting the penetration of fluid and aggressive ions, which transforms the interfacial failure mode from simple adhesive peeling to a more stable mechanical interlocking resistance. Thus, the thick corrosion products layer formation is slowed down and the risk of H_2_-induced interfacial delamination is reduced. This mechanism is favored for maintaining the structure of the composite as well as for providing the homogeneous corrosion morphology of the Mg-F-C/PLA sheet during degradation.

As illustrated in Figure 8c, the Mg/PLA composite provides a highly favorable osteogenic microenvironment. The controllable degradation leads to a moderate and continuous release of Mg ions, which can greatly promote the survival and proliferation of MC3T3-E1 osteoblasts. To sum up, the combination of two modifications on the Mg-F-C/PLA sheet can effectively improve the bonding strength between Mg and PLA through physical entanglement effects. Therefore, the mechanical durability, long-term degradation stability, and cytocompatibility are effectively improved, making the composite a very attractive candidate for use in load-bearing orthopedic fixation devices [34].

## 5. Conclusions

This work proposes a dual interfacial enhancement strategy combining HF fluorination and laser perforation to strengthen Mg/PLA composites via both chemical bonding and mechanical interlocking.

Firstly, finite element modeling and experimental results show that a pore volume fraction of 2.5% yields optimal bending performance. The Mg-F-C/PLA composite achieves a maximum bending strength of 90.5 MPa and a maximum strain of 24.1%, significantly outperforming pure PLA.

Secondly, during in vitro degradation, the Mg-F-C/PLA composite retained approximately 85% of its initial bending strength after 6 weeks. The gradual changes in pH and Mg^2+^ release confirm enhanced degradation stability. Biocompatibility tests indicated high cell viability of all treatment groups (>90%) in 100% extract solution.

Finally, the dual surface treatment of the composite sheets established mechanical interlocking to suppress interfacial delamination and delayed degradation of the MgF_2_ protective layer. These synergistic effects of the Mg-F-C/PLA composite collectively delay strength loss and maintain structural integrity, providing a practical design strategy for fracture fixation devices requiring sustained mechanical stability in clinical settings.

## Data Availability

The original contributions presented in this study are included in the article. Further inquiries can be directed to the corresponding authors.

## References

[1] Kim Y., Kwak J., Lim M., Lim S.Y., Chae S., Ha S.-J., Won Y.-W., Kim H.-O., Lim K.S. (2025). Advances in PCL, PLA, and PLGA-Based Technologies for Anticancer Drug Delivery. Pharmaceutics.

[2] Jha S., Akula B., Enyioma H., Novak M., Amin V., Liang H. (2024). Biodegradable Biobased Polymers: A Review of the State of the Art, Challenges, and Future Directions. Polymers.

[3] Lata S., Mondal S., Mishra R., Priya S., Deshwal R.K., Thapliyal S., Talukdar N., Dhasmana A., Rustagi S., Bora J. (2025). Advancements in biodegradable implants: Evaluation in large animal surgical models. Ann. Med. Surg..

[4] Subhedar P., Padmanabhan D., Agrawal R. (2025). Biomedical Applications of Polycaprolactone (PCL) Composites: Structure, Properties, and Future Prospects. J. Res. Updates Polym. Sci..

[5] Luque-Agudo V., Casares-López J.M., González-Martín M.L., Gallardo-Moreno A.M., Hierro-Oliva M. (2023). PLA-Mg film degradation under in vitro environments supplemented with glucose and/or ketone bodies. Polym. Test..

[6] García-Sobrino R., Muñoz M., Rodríguez-Jara E., Rams J., Torres B., Cifuentes S.C. (2023). Bioabsorbablecomposites based on polymeric matrix (PLA and PCL) reinforced with magnesium (Mg) for use in bone regeneration therapy: Physicochemical properties and biological evaluation. Polymers.

[7] Azar M.G., Wiesnerova L., Dvorakova J., Chocholata P., Moztarzadeh O., Dejmek J., Babuska V. (2023). Optimizing PCL/PLGA Scaffold Biocompatibility Using Gelatin from Bovine, Porcine, and Fish Origin. Gels.

[8] Wang S., Lu M., Cao Y., Tao Z., Sun Z., Liu X., Liu J., Liu S. (2023). Degradative polylactide nanofibers promote M2 macrophage polarization via STAT6 pathway in peritendinous adhesion. Compos. Part B.

[9] Feng P., Jia J., Liu M., Peng S., Zhao Z., Shuai C. (2021). Degradation mechanisms and acceleration strategies of poly (lactic acid) scaffold for bone regeneration. Mater. Des..

[10] Sun Y., Zheng Z., Wang Y., Yang B., Wang J., Mu W. (2022). PLA composites reinforced with rice residues or glass fiber—A review of mechanical properties, thermal properties, and biodegradation properties. J. Polym. Res..

[11] Ali W., Mehboob A., Han M.-G., Chang S.-H. (2020). Novel biodegradable hybrid composite of polylactic acid (PLA) matrix reinforced by bioactive glass (BG) fibres and magnesium (Mg) wires for orthopaedic application. Compos. Struct..

[12] Wang X., Zhao Y., Dong Q., Chu C., Xue F., Wang C., Bai J. (2025). Stimuli-responsive magnesium-based materials for biomedical applications: A review. Responsive Mater..

[13] Zhou X., Xin J., Wang C., Qian K., Tao X., Ba Z., Xue F., Bai J., Mallia B., Dong Q. (2025). Advances toward self-healing coatings on Mg alloys for active corrosion protection. J. Magnes. Alloys.

[14] Zhao C., Wu H., Ni J., Zhang S., Zhang X. (2017). Development of PLA/Mg composite for orthopedic implant: Tunable degradation and enhanced mineralization. Compos. Sci. Technol..

[15] Ali F., Al Rashid A., Kalva S.N., Koç M. (2023). Mg-Doped PLA Composite as a Potential Material for Tissue Engineering—Synthesis, Characterization, and Additive Manufacturing. Materials.

[16] Lee H., Shin D.Y., Bang S.-J., Han G., Na Y., Kang H.S., Oh S., Yoon C.-B., Vijayavenkataraman S., Song J. (2024). A strategy for enhancing bioactivity and osseointegration with antibacterial effect by incorporating magnesium in polylactic acid based biodegradable orthopedic implant. Int. J. Biol. Macromol..

[17] Zhang H.Y., Jiang H.B., Kim J.-E., Zhang S., Kim K.-M., Kwon J.-S. (2020). Bioresorbable magnesium-reinforced PLA membrane for guided bone/tissue regeneration. J. Mech. Behav. Biomed. Mater..

[18] Bakhshi R., Mohammadi-Zerankeshi M., Mehrabi-Dehdezi M., Alizadeh R., Labbaf S., Abachi P. (2023). Additive manufacturing of PLA-Mg composite scaffolds for hard tissue engineering applications. J. Mech. Behav. Biomed. Mater..

[19] Cai H., Zhang Y., Meng J., Li X., Xue F., Chu C., Tao L., Bai J. (2018). Enhanced fully-biodegradable Mg/PLA composite rod: Effect of surface modification of Mg-2Zn wire on the interfacial bonding. Surf. Coat. Technol..

[20] Abdeljawad M.B., Carette X., Mincheva R., Odent J., Raquez J.-M. (2021). Interfacial compatibilization of PLA and Mg in composites for bioresorbable bone implants. IOP Conf. Ser. Mater. Sci. Eng..

[21] Lapczyk I., Hurtado J.A. (2007). Progressive damage modeling in fiber-reinforced materials. Compos. Part A.

[22] Liu C., Du D., Li H., Hu Y., Xu Y., Tian J., Tao G., Tao J. (2016). Interlaminar failure behavior of GLARE laminates under short-beam three-point-bending load. Compos. Part B.

[23] (2015). Standard Test Method for Flexural Properties of Polymer Matrix Composite Materials, 1st ed..

[24] Ye J., Miao B., Xiong Y., Guan Y., Lu Y., Jia Z., Wu Y., Sun X., Guan C., He R. (2025). 3D printed porous magnesium metal scaffolds with bioactive coating for bone defect repair: Enhancing angiogenesis and osteogenesis. J. Nanobiotechnol..

[25] Fattah-alhosseini A., Chaharmahali R., Rajabi A., Babaei K., Kaseem M. (2022). Performance of PEO/Polymer Coatings on the Biodegradability, Antibacterial Effect and Biocompatibility of Mg-Based Materials. J. Funct. Biomater..

[26] Zhai C., Sun Q., Ma A., Wang W., Zhu T., Zhang Y., Yu T., Yang Z., Lan J., Wang Z. (2025). Preparation and characterization of corrosion-resistant MgF_2_-PDA-EGCG coatings on AZ31B magnesium alloy with antibacterial and osteoinductive properties. Ceram. Int..

[27] Wang C., Dong Q., Shao Y., Wu Y., Zhao Y., Yin M., Mei D., Chu C., Xue F., Bai J. (2026). Probing interfacial electrochemical processes in metal biodegradation via in operando pH microscopy: A review with perspectives. TrAC Trends Anal. Chem..

[28] Ali W., Echeverry-Rendón M., Kopp A., González C., LLorca J. (2023). Effect of surface modification on interfacial behavior in bioabsorbable magnesium wire reinforced poly-lactic acid polymer composites. npj Mater. Degrad..

[29] Mei D., Li Y., Tian Y., Zhang Q., Liu M., Zhu S., Wang L., Guan S. (2024). The effect of selected corrosion inhibitors on localized corrosion of magnesium alloy: The expanded understanding of “inhibition efficiency”. Corros. Sci..

[30] Cai H., Li X., Chu C., Xue F., Guo C., Dong Q., Bai J. (2019). Insight into the effect of interface on the mechanical properties of Mg/PLA composite plates. Compos. Sci. Technol..

[31] Wang Y., Zhang W., Hua Y., Mao Y., Xv Q., Zhong C., Cao S., You D., Wang X. (2025). Inorganic-organic composite protective coating for biodegradable metal ureteral stents. Mater. Chem. Phys..

[32] Liu M., Mei D., Zhu S., Blawert C., Zheludkevich M.L., Guan S., Lamaka S.V. (2025). The external stress-assisted corrosion behavior of Mg alloys for biomedical applications. Corros. Sci..

[33] Cai H., Li X., Xue F., Chu C., Guo C., Bai J., Zhang X. (2021). In Vitro Study on Cytocompatibility of Mg Wire/Poly(Lactic Acid) Composite Rods. J. Mater. Eng. Perform..

[34] Ali W., Echeverry-Rendón M., Kopp A., González C., LLorca J. (2021). Strength, corrosion resistance and cellular response of interfaces in bioresorbable poly-lactic acid/Mg fiber composites for orthopedic applications. J. Mech. Behav. Biomed. Mater..

